# Predicting the side effects of drugs using matrix factorization on spontaneous reporting database

**DOI:** 10.1038/s41598-021-03348-y

**Published:** 2021-12-14

**Authors:** Kohei Fukuto, Tatsuya Takagi, Yu-Shi Tian

**Affiliations:** Graduate School of Pharmaceutical Sciences, Osaka University, 1-6 Yamadaoka, Suita City, Osaka, 565-0871 Japan

**Keywords:** Data mining, Machine learning, Adverse effects

## Abstract

The severe side effects of some drugs can threaten the lives of patients and financially jeopardize pharmaceutical companies. Computational methods utilizing chemical, biological, and phenotypic features have been used to address this problem by predicting the side effects. Among these methods, the matrix factorization method, which utilizes the side-effect history of different drugs, has yielded promising results. However, approaches that encapsulate all the characteristics of side-effect prediction have not been investigated to date. To address this gap, we applied the logistic matrix factorization algorithm to a database of spontaneous reports to construct a prediction with higher accuracy. We expressed the distinction in the importance of drug-side effect pairs by a weighting strategy and addressed the cold-start problem via an attribute-to-feature mapping method. Consequently, our proposed model improved the prediction accuracy by 2.5% and efficiently handled the cold-start problem. The proposed methodology is expected to benefit applications such as warning systems in clinical settings.

## Introduction

Drugs with severe side effects are fatal to patients and damage pharmaceutical companies financially. Drug safety information is typically evaluated using data from non-clinical studies and clinical trials. However, due to the limited number of patients and lower diversity of patient participation in clinical trials compared to those in actual use, it is fairly common for unknown side effects to be identified after a drug is launched.

Approaches for predicting the side effects of clinical drugs can be broadly divided into chemical features such as drug structures; biological features such as target proteins, transporters, and enzymes; and phenotypic features such as side effects and therapeutic indications. Previous studies have explored algorithms that are best suited to these approaches, such as the use of sparse canonical correlation analysis based on the chemical structure of drugs; canonical correlation analysis and kernel regression based on chemical structures of drugs and target proteins; and logistic regression, naïve Bayes, k-nearest neighbor method, random forest, and support vector machines (SVM) based on chemical, biological, and phenotypic features^1–3^. In previous research, SVM showed the highest potential, and the phenotypic features were the most influential in acquiring predictions^3^. In another study, side-effect prediction was considered a multi-label prediction task; accordingly, a k-nearest neighbor-based multi-label learning method was proposed^4^.

The premise of predicting an unknown side effect based on known side effects (using the phenotypic features) is inspired by recommender systems, which are commonly utilized in e-commerce websites to suggest products to users based on their past ratings and behavioral history. To date, predictive pharmacosafety networks (PPNs), which are used to construct a network of drugs and side effects, and matrix factorization (MF), one of the most basic algorithms in recommender systems, have been applied to predict unknown side effects^5,6^. Furthermore, MF regularized by drug and side-effect similarities has also been investigated for similar purposes^7,8^.

However, these algorithms do not address several aspects of side-effect prediction. First, the known side effect information is implicit feedback, that is, if a side effect for a drug has not been reported, then an association between them either does not exist or has not been observed yet. However, MF models are typically designed for explicit feedback data. Second, previous studies have not adequately accounted for the differences in weights among known drug-side effect pairs, apart from Xie and Poleksic^8^, where they are all set to 1, and configuring these weights may prove pivotal in improving the prediction results. Finally, recommender systems are known to be afflicted by the cold-start problem, wherein the system is unable to provide suitable predictions for drugs with very few known side effects, and no precedent has been set for this in side-effect prediction^9,10^.

Additionally, previous studies use the Side Effect Resource (SIDER), an aggregated database comprising official documents and package inserts, for model training and evaluation^6–8,11^. However, the latency in the occurrence of a side effect and updation of pertinent documentation may render the database obsolete for predicting side effects, which typically warrants real-time information. Therefore, we developed a custom dataset for this study derived from the FDA Adverse Event Reporting System (FAERS), a database of spontaneous adverse drug reaction reports maintained by the United States Food and Drug Administration (FDA).

Here, we utilized the logistic matrix factorization (Logistic MF) model^12^, a modified MF model with implicit feedback, to predict severe side effects of clinical drugs more effectively based on a custom dataset derived from the FAERS database. We also simulated a cold-start scenario, investigated its impact, and explored attribute-to-feature mapping as a solution^13^.

## Methods

**Figure 1:**
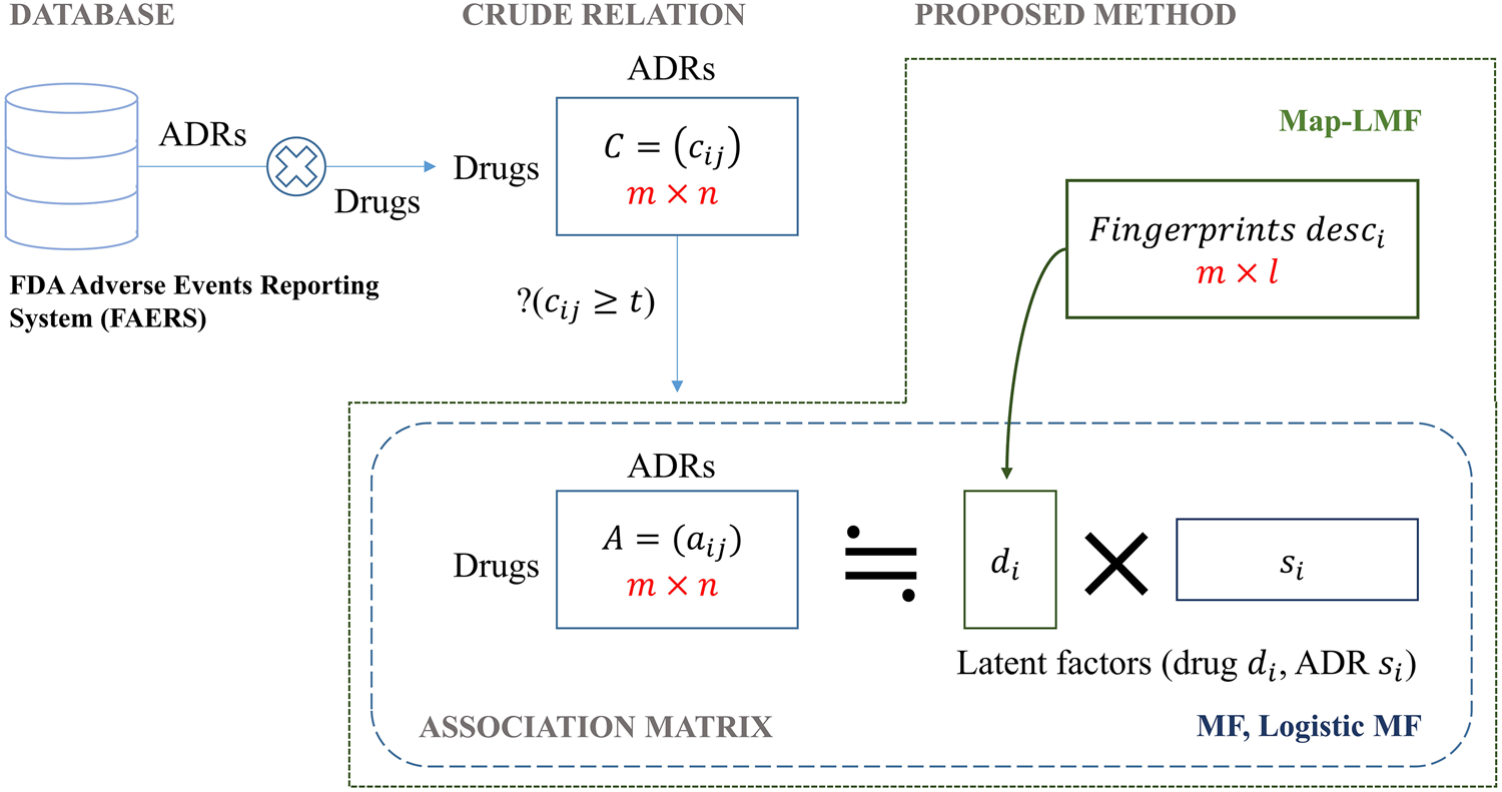
Flow chart of this study.

The flowchart for this study is shown in Fig. 1.

### Dataset

We downloaded the FAERS database, which stores spontaneous reports from healthcare professionals, patients, and pharmaceutical companies, from 2004 Q1 through 2019 Q2. The *DRUG* and *REAC* tables, in particular, were used to compile drug names and their corresponding side effects. A dataset representing associations between 1127 drugs and 5237 side effects, including 68 severe side effects, was created (see SI Appendix).

### Prediction models

#### Matrix factorization

The classic MF algorithm with explicit feedback has been extensively applied to movie rating predictions and other recommender systems. This method and its variants have previously been used for side-effect predictions^6,7^.

Let $m$ denote the number of drugs and $n$ represent the number of side effects. The number of reports for all drug-side effect pairs is represented by the $m \times n$ matrix, $C = \left( {c_{ij} } \right)$, where $c_{ij}$ is the number of times drug $i$ is reported as the primary suspect for side effect $j$. When we compared $c_{ij}$ with a threshold of occurrence $t$, we obtained a matrix $A = \left( {a_{ij} } \right)$ that represents the association of all drug-side effect pairs given as follows.

$$a_{ij} = \left\{ {\begin{array}{*{20}l} {1,\,\,\,\, c_{ij} \ge t} \\ {0,\,\,\,\, c_{ij} < t} \\ \end{array} } \right.$$

The larger the threshold of occurrence, the more likely it is that true drug-side effect associations are overlooked, and the smaller the threshold, the more likely it is that noise in the dataset is labeled as meaningful signals. Thus, we configured the threshold value as $t = 3$ to reduce the false positives for this study in compliance with the conventions in the signal detection field^14,15^. The influence of the threshold was also evaluated by shifting $t$ from 3 to 5.

MF assumes that each drug and side effect has latent factors of dimension $k$. Let $d_{i}$ denote the latent factor vector of drug $i$ and $s_{j}$ of side effect $j$, then $a_{ij}$ can be estimated as

$$\hat{a}_{ij} = d_{i}^{T} s_{j} + b_{i} + b_{j}$$

where $b_{i}$ and $b_{j}$ are the bias terms for drug $i$ and side effect $j$ respectively^16^.

Latent factors are learned by minimizing the squared error as:

$$\mathop {\min }\limits_{D, S} \mathop \sum \limits_{{\left( {i,j} \right) \in A}} \left( {a_{ij} - \hat{a}_{ij} } \right)^{2} + \lambda \left( ||{d_{i}||^{2} + ||s_{j}||^{2} } \right)$$

where $D$ is an $m \times k$ matrix with row $i$ being $d_{i}$, and $S$ is an $n \times k$ matrix with row $j$ being $s_{j}$. The second term in the loss function is the L2 penalty term for the latent factors to prevent overfitting. $\lambda$ is a hyperparameter that controls the degree of regularization.

However, this method has two shortcomings. First, the number of reported side effects can be regarded as implicit feedback for the true drug-side effect associations; hence, there is no distinction between the negative and unobserved examples in $A$, implying that the corresponding zero entries are potential positive examples. However, the model learns these zero entries as is, thereby reducing its efficiency in predicting missing side effects. Second, the model does not consider differences in the importance or weight of the associations between drugs and side effects.

#### Logistic matrix factorization

Logistic MF modifies the MF schema for the implicit feedback data^12^. Assuming that the objective variable in the implicit feedback data is binary, Logistic MF employs the sigmoid function, $\sigma$, to supply predictions. Then $a_{ij}$ is computed as:

$$\hat{a}_{ij} = \sigma \left( {d_{i}^{T} s_{j } + b_{i} + b_{j} } \right)$$

Latent factors are learned by minimizing the log loss as:

$$\mathop {\min }\limits_{D,S} - \mathop \sum \limits_{{\left( {i,j} \right) \in D}} w_{ij} \left\{ {a_{ij} \log \hat{a}_{ij} + \left( {1 - a_{ij} } \right)\log \left( {1 - \hat{a}_{ij} } \right)} \right\} + \lambda \left( ||{d_{i}||^{2} + ||s_{j}||^{2} } \right)$$

where $w_{ij}$ corresponds to the weight of each drug-side effect pair.

In a previous study^12^, $c_{ij} = t, t = 1$ is the preconfigured threshold, and $w_{ij} = \alpha c_{ij}$ and $w_{ij} = 1 + \alpha \log (1 + c_{ij} /\varepsilon )$ were considered examples of the weighting functions, where $\alpha$ was a hyperparameter. However, these weighting functions vary depending on the characteristics of the problem. Hence, for this study, we configured $c_{ij} = t, t = 3$. Assuming that the effect of the number of reports on the weights is not linear but grows logarithmically, we used the following weighting function:

$${ }w_{ij} = \left\{ {\begin{array}{*{20}l} {1 + \alpha \log \left( {1 + c_{ij} } \right),\,\,\,\, c_{ij} \ge t} \\ {\beta ,\,\,\,\, c_{ij} < t} \\ \end{array} } \right.$$

where $\beta$ is another hyperparameter used to reduce the impact of negative examples on the overall loss function to account for implicit feedback. It should be noted that the logarithmic assumption is not a unique choice for this problem. Other functions whose output values do not change significantly when the input values are large enough may also exhibit similar potentials. As expected, the linear weighting function is not suitable here (data not shown).

#### Attribute-to-feature mapping

Attribute-to-feature mapping is known to improve the prediction accuracy in cold-start scenarios by learning the mapping function of the user or item attributes to latent factor vectors^13^. In cold-start problems associated with side-effect predictions, adequate information of the side effects for a particular drug is not available, causing the model to learn the latent factor vectors incorrectly. In this case, estimating latent factors from secondary data, such as drug structures, may help improve prediction accuracy.

The k-nearest neighbor and linear mapping algorithms have previously been proposed to map attributes to latent factors, eliciting superior results when the latter algorithm is optimized for the final evaluation metric rather than the squared error, except for when the dimension of the attributes is extremely high^13^. Here, a linear mapping from attributes to latent factors of drugs is expressed as:

$$\hat{d}_{i} = M^{T} desc_{i}$$

where $desc_{i}$ is the attribute of drug $i$, and $M$ is the learnable parameter matrix of the mapping function with the shape of ($n$, $k$), where $n$ is the dimension of the drug attribute and $k$ is the dimension of the latent factors.

For drug attributes, RDKit molecular descriptors^17^ and extended-connectivity fingerprints (ECFP)^18^ were used. The 2048-bit fingerprints generated by the ECFP were reduced to 100 dimensions using kernel principal component analysis (KPCA)^19^. The hyperparameters of the KPCA were determined by conducting a grid search on the validation set.

### Experiment

#### Data preparation

We attempted to construct MF and Logistic MF models for side-effect prediction and investigated the impact of the cold-start problem. The cold-start scenario was simulated by removing some of the known side effects of the drugs used for model evaluation. However, if we randomly split all drug and side effect pairs into training, validation, and test sets as in the typical evaluation scheme of MF, at least one drug and side effect pair for most drugs will be included in the test set. Thus, removing some of the training pairs of these drugs will significantly reduce the amount of training data, resulting in an unrealistic situation. Therefore, we adopted a unique data-splitting strategy to ensure that the simulation did not affect the model training.

**Figure 2:**
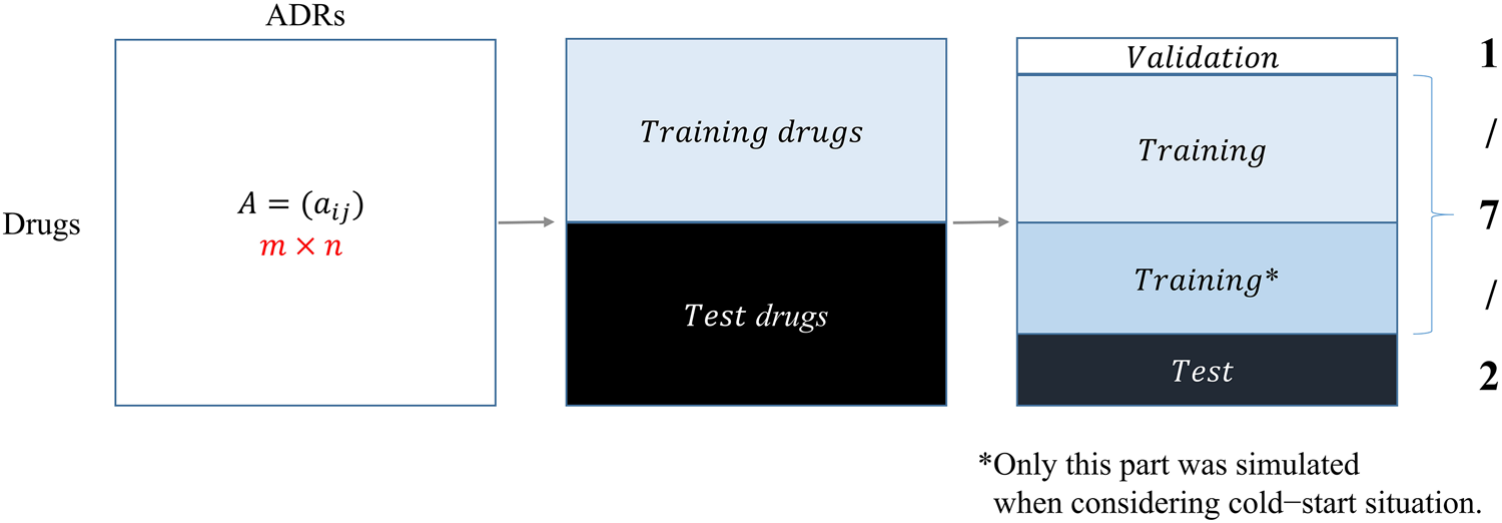
The method for split training and test sets.

The dataset leading up to 2015 Q3 was employed in this study. Drugs were randomly split in half to procure the training and test drugs, 20% of the training drug and side effect pairs were set aside for validation, while the rest were used for training, and 40% of test drug and side effect pairs were used for testing, while the rest were used for training. Overall, 70% of all the drug-side effect pairs were used for training, 10% for validation, and 20% for testing. When considering the cold-start situation, only the side effect information in the training sets from the test drugs was removed. In contrast, the known side effects of the training drugs remained the same (Fig. 2).

#### Evaluation metric

The area under the precision-recall curve (PR-AUC) was the primary evaluation metric for each side effect. All training data pairs were used to calculate the loss function during training, but the average PR-AUC of severe side effects was used for early stopping. The dataset was partitioned five times using different random seeds, and the mean and standard deviation of the evaluation metrics were computed.

#### Hyperparameter search

A grid search was conducted to locate the hyperparameters with the highest evaluation metric in the validation set (Table 1). The experiment was repeated five times, and the hyperparameters obtained in the first repetition were fixed for the following cycles. The latent factor parameters were regularized using λ, while α and β were used to adjust the positive and negative example weights. The latent factor dimensionality was fixed at 100^7^. The number of training epochs was determined by early stopping with PR-AUC in the validation set. The initial learning rate was set to 0.01 and was scheduled to decrease at a fixed rate of 0.1 whenever the PR-AUC value dipped in the validation set to avoid local optimal solutions. The Adam optimizer was applied to the loss function^20^.

**Table 1:** List of hyperparameters and their range in the grid search.

	Hyperparameter	Range
Logistic MF	λ	[1.0 × 10^–4^, 5.0 × 10^–4^, 1.0 × 10^–3^, 5.0 × 10^–3^, 1.0 × 10^–2^]
	α	[0, 1, 2, 5, 10, 15]
	β	[0.2, 0.4, 0.6, 0.8, 1.0]
MF	λ	[1.0 × 10^–4^, 5.0 × 10^–4^, 1.0 × 10^–3^, 5.0 × 10^–3^, 1.0 × 10^–2^]
FGRMF	λ	[1.0 × 10^–5^, 5.0 × 10^–5^, 1.0 × 10^–4^, 5.0 × 10^–4^, 1.0 × 10^–3^]
	μ	[1.0 × 10^–4^, 5.0 × 10^–4^, 1.0 × 10^–3^, 5.0 × 10^–3^, 1.0 × 10^–2^]
SVM	C	[1.0 × 10^–5^, 1.0 × 10^–4^, 1.0 × 10^–3^, 1.0 × 10^–2^, 1.0 × 10^0^, 1.0 × 10^1^, 1.0 × 10^2^]
	kernel	[“linear”, “poly”, “rbf”]

#### Comparison with other models

To evaluate performance, we compared our proposed Logistic MF model and several previously reported models, including MF as mentioned earlier, feature-derived graph regularized matrix factorization (FGRMF)^7^, and support vector machine (SVM)^3^. For FGRMF, PubChem fingerprints were used per the suggestion in a previous report^7^, and for SVM, phenotypic features (other known side effects vector) were used as input features. In the previous SVM model^3^, the indication feature was also used as a phenotypic feature; however, we did not include it to make a fair comparison with other models using only known side effect information.

#### Comparison with an external database

We also used the Side Effect Resource (SIDER) database^11^ to evaluate model performance. The SIDER database contained associations between marketed drugs and their side effects. However, frequency information was provided for only 39.9% of drug-side effect pairs, which is insufficient for use in the weighting functions of Logistic MF. Thus, we retrieved the corresponding frequency information for each pair from the FAERS. The reports in FAERS until the release date of SIDER 4.1 (21 Oct, 2015) were used to acquire frequency data to ensure that the periods in both data sources were consistent. Other procedures were the same as those mentioned above.

#### Cold-start simulations

As stated earlier, the cold-start problem is a major handicap for MF and Logistic MF. We simulated a cold-start scenario, that is, reducing the number of known side effects of the test drugs, and investigated its impact on the prediction performance of the proposed model. We randomly removed training data for a test drug in a defined *test_delete_ratio* and reported the evaluation metrics of the test set at different *test_delete_ratio*s*.* The deletion probability was weighted based on the number of known side effects.

We applied attribute-to-feature mapping to our model, represented by Map-LMF, for the cold-start scenario.

### Consent to publish

All the authors agree to publish.

## Results and discussion

### Performance of Logistic MF model

Table 2 highlights the mean and standard deviations of PR-AUCs in the test set for data up to 2015 Q3 for the MF, Logistic MF, and FGRMF models. For a concise view of our clinical interests, we showed the results of all 68 severe side effects and three representative diseases – Stevens-Johnson syndrome (SJS), low platelet counts (LPT), and neuroleptic malignant syndrome (NMS). We showed the results for other severe side effects in Table S1. Logistic MF exhibited a PR-AUC of 0.812 ± 0.021 and outperformed the other models. The mean PR-AUC of Logistic MF improved by 2.5% compared to that of MF. Despite the large standard deviation attributed to a limited number of positive examples in the test set, the sigmoid and weight functions consistently demonstrated superior prediction performance. The optimal hyperparameters used here were λ = 0.005 for MF, λ = 0.005, α = 10, and β = 0.8 for Logistic MF, µ = 0.0005, and λ = 0.0001 for FGRMF. We trained the SVM on each side effect independently, and the optimal hyperparameters varied depending on the side effect.

**Table 2:** PR-AUC of test sets for Logistic MF and other models.

	Mean (68 ADRs)	SJS	LPT	NMS
Logistic MF	0.812 $\pm$ 0.021	0.865 $\pm$ 0.017	0.948 $\pm$ 0.012	0.771 $\pm$ 0.089
MF	0.787 $\pm$ 0.018	0.877 $\pm$ 0.016	0.941 $\pm$ 0.021	0.685 $\pm$ 0.078
FGRMF	0.752 $\pm$ 0.014	0.800 $\pm$ 0.023	0.821 $\pm$ 0.041	0.699 $\pm$ 0.099
SVM	0.763 $\pm$ 0.018	0.794 $\pm$ 0.106	0.938 $\pm$ 0.038	0.755 $\pm$ 0.118

### Impact of the thresholds and regularization

**Figure 3:**
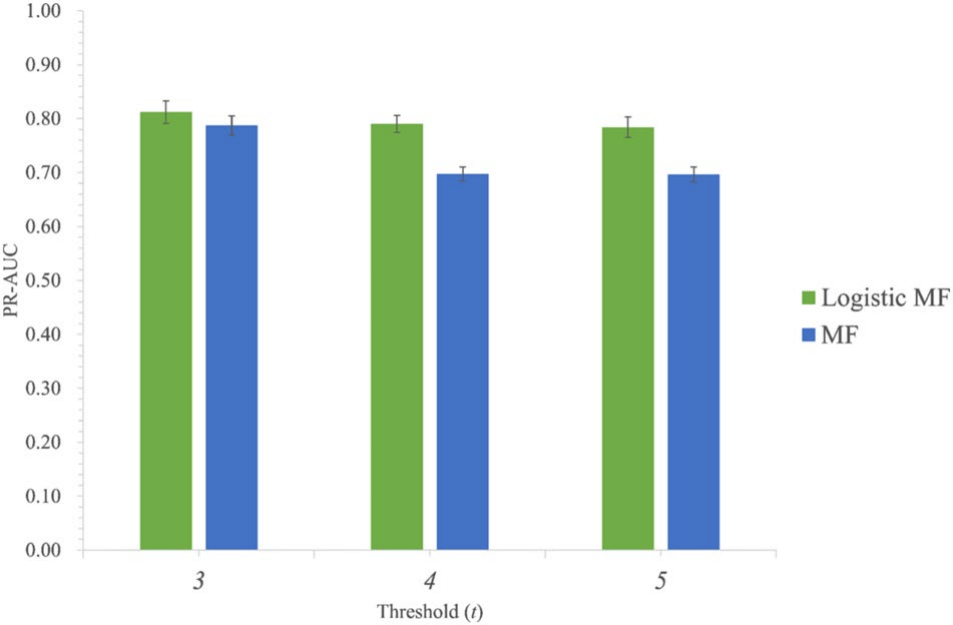
The change of PR-AUC when using different thresholds.

To confirm the impact of thresholds on the result, we changed the threshold values in the dataset creation and compared the MF and Logistic MF models using the altered dataset. We conducted these experiments using the same procedure mentioned earlier. In Fig. 3, we showed the results with threshold values $t =$ 3, 4, and 5. Logistic MF outperformed MF under all threshold settings in mean PR-AUC, which indicated that the acquired result was independent of the thresholds and that this method is robust. We also confirmed that L1 regularization is not as effective as L2 regularization (Fig. S1).

### External validation using future data

We evaluated the viability and robustness of the proposed model using data from the 2015 Q4 onwards. To achieve this, we randomly split data pairs up to 2015 Q3, where 10% was used as the validation set. All models were trained and the model output for drug-side effect pairs with negative labels in the training set (i.e., the pairs occurring less than three times by 2015 Q3) were obtained. The PR-AUCs were then computed using future labels. Table 3 summarized the results, and we listed those of other severe side effects in Table S2.

**Table 3:** PR-AUC of the external tests for Logistic MF and other methods.

	Mean (68 ADRs)	SJS	LPT	NMS
Logistic MF	0.297 $\pm$ 0.001	0.293 $\pm$ 0.013	0.243 $\pm$ 0.013	0.234 $\pm$ 0.014
MF	0.291 $\pm$ 0.008	0.275 $\pm$ 0.031	0.246 $\pm$ 0.025	0.226 $\pm$ 0.018
FGRMF	0.293 $\pm$ 0.002	0.277 $\pm$ 0.010	0.264 $\pm$ 0.018	0.251 $\pm$ 0.029
SVM	0.195 $\pm$ 0.005	0.133 $\pm$ 0.071	0.149 $\pm$ 0.017	0.078 $\pm$ 0.067

External validation results again favor our Logistic MF model over other models in predicting side effects more accurately (Table 3). Please note that the PR-AUC values in Tables 2 and 3 cannot be compared directly, owing to the difference in the number of positive examples in the validation schemes, affecting the PR-AUC values. However, the difference in these values is significant, indicating, employing a random split on data generated in a time-series manner may invoke an overly optimistic evaluation of the prediction performance in all models.

### External validation using the SIDER database

We presented the results for the SIDER database in Tables 4 and S3. Logistic MF still outperformed MF, suggesting Logistic MF improved the performance of MF not only for the FAERS data but also for other databases. However, FGRMF had a higher mean PR-AUC (0.481 $\pm$ 0.012) than MF and Logistic MF. This result may be attributed to inconsistency between SIDER labels and FAERS frequency, as the former is extracted from public documents such as package inserts, and the latter is directly taken from spontaneous reports. This indicated that accurate frequency information might be needed to take advantage of Logistic MF. SVM performed best among models. SVM was trained on individual side effects, while MF-based models were trained for all side effects at once. The SIDER dataset has less correlated labels compared to the FAERS dataset. Thus individually trained SVM performed better for the SIDER dataset. However, employing SVM to predict side effects has several drawbacks. First, SVM must be trained separately for each side effect. Tuning the hyperparameters for all models needs much more time than tuning a single model for all side effects with MF-based models. Second, SVM cannot handle cold-start problems. The flexibility of MF-based models allows us to apply attribute-to-feature mapping to handle the cold-start situation effectively. Considering these aspects, MF-based models can still be a good choice for real-world side effect prediction.

**Table 4:** PR-AUC of test set in SIDER for Logistic MF and other methods.

	Mean	SJS	LPT	NMS
Logistic MF	0.462 $\pm$ 0.015	0.540 $\pm$ 0.085	0.777 $\pm$ 0.013	0.658 $\pm$ 0.160
MF	0.445 $\pm$ 0.012	0.453 $\pm$ 0.070	0.742 $\pm$ 0.056	0.701 $\pm$ 0.147
FGRMF	0.481 $\pm$ 0.012	0.494 $\pm$ 0.056	0.769 $\pm$ 0.030	0.722 $\pm$ 0.125
SVM	0.551 $\pm$ 0.015	0.689 $\pm$ 0.069	0.869 $\pm$ 0.032	0.681 $\pm$ 0.113

### Cold-start problem: simulated results

**Figure 4:**
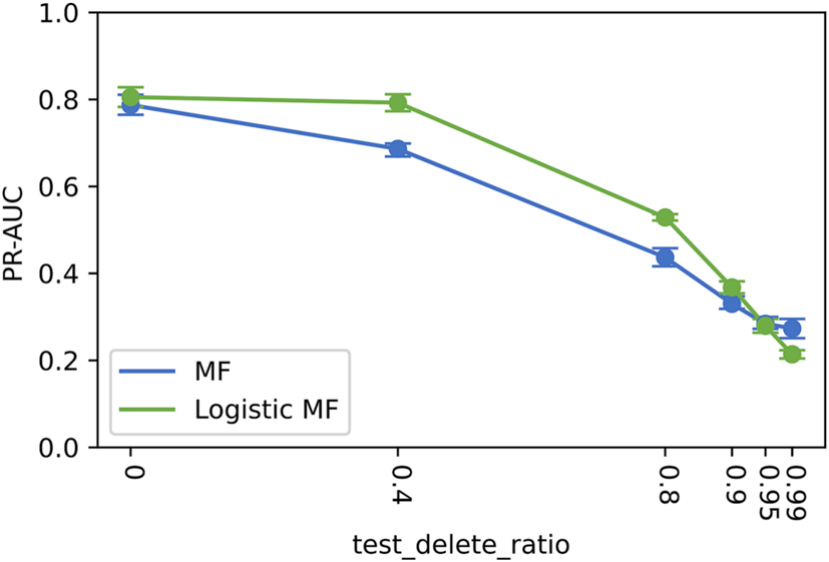
PR-AUC of test sets with varying number of known side effects.

We showed the simulated results for the logistic MF in treating the cold-start problem in Fig. 4. We also showed the simulation results for MF as a reference to confirm the effect of weights in these settings. The PR-AUC decreased significantly with fewer known side effects, suggesting that the prediction accuracy of our model deteriorated when test drug information was insufficient, as may be the case with drugs in the early stages of development or clinical trials.

### Effect of attribute-to-feature mapping

The PR-AUCs of the Logistic MF and Map-LMF models for varying numbers of known side effects are presented in Table 5.

**Table 5:** Test PR-AUC for Logistic MF and Map-LMF with varying number of known side effects.

Test_delete_ratio	0.80	0.90	0.95	0.99
Logistic MF	0.550 $\pm$ 0.009	0.377 $\pm$ 0.008	0.286 $\pm$ 0.011	0.235 $\pm$ 0.010
Map-LMF (RDKit)	0.309 $\pm$ 0.009	0.310 $\pm$ 0.010	0.308 $\pm$ 0.010	0.308 $\pm$ 0.010
Map-LMF (ECFP)	0.357 $\pm$ 0.019	0.357 $\pm$ 0.020	0.358 $\pm$ 0.020	0.359 $\pm$ 0.019

Predicting the latent factor vectors using ECFP as the drug attribute improved the prediction accuracy under cold-start settings. The prediction accuracy of Map-LMF exceeded that of Logistic MF by 2.2% and 7.3% at *test_delete_ratio* = 0.95 and 0.99, with RDKit descriptors, and by 7.2% and 12.4% at *test_delete_ratio* = 0.95 and 0.99 with ECFP. As previously established, inadequate information on the known side effects of a test drug adversely affects the prediction accuracy. Therefore, the latent factors we estimated from the chemical structure of the drugs provided better predictions.

By using estimated latent factors from Map-LMF when side effect information is insufficient, we maintained predictive performance of Logistic MF for drugs with abundant known side effects and alleviated the performance drop of Logistic MF in drugs with less known side effects. Flexibility in replacing drug latent factors with estimated latent factors made it easy to combine Map-LMF with Logistic MF.

## Conclusion

Drugs with severe side effects endanger patients and pharmaceutical companies. Therefore, an effective methodology needs to be investigated to predict these side effects and, in turn, ascertain patient safety and efficient drug development. MF has previously been utilized for prediction of side-effects. We consolidated the available knowledge on MF and its shortcomings, such as its inability to handle implicit feedback and cold start problems, and identified Logistic MF as an efficient model to meet our objectives. The results affirmed that our proposed model improved the overall prediction accuracy by 2.5% and produced superior performance in the cold-start settings using attribute-to-feature mapping by at most 12.4%.

The limitations of this study are: We could not determine whether all drugs from the FAERS database were included in the final dataset during data pre-processing because of incomplete mapping between drug names and their structures. Furthermore, the preconfigured threshold value for forging drug-side effect associations may have overlooked the possibility of mislabeled drugs caused by noise in the spontaneous reports database. In future, we intend to incorporate a signal detection criterion to extract drug-side effects pairs from the reports database more accurately and find feasible solutions to the other drawbacks identified.

## Supplementary Information

Supplementary Information.

## Data Availability

This study analyzed the FAERS database, which can be obtained from the US FDA. The codes used in the current study are available at https://github.com/ykskks/Matrix-Factorization-for-Drug-Side-Effect-Prediction.

## Abbreviations

ECFP: Extended-connectivity fingerprints
FAERS: The FDA Adverse Event Reporting System
FDA: The United States Food and Drug Administration
KEGG: Kyoto Encyclopedia of Genes and Genomes
KPCA: Kernel principal component analysis
MF: Matrix factorization
MHLW: The Ministry of Health Labour and Welfare
PPN: Predictive pharmacosafety networks
PR-AUC: Area under the precision-recall curve
SIDER: Side effect resource
SVM: Support vector machine
