# Supplementary material for: Predicting the side effects of drugs using matrix factorization on spontaneous reporting database
Source: Sci Rep. 2021 Dec 14;11:23942. doi: 10.1038/s41598-021-03348-y (PMC8671428; doi:10.1038/s41598-021-03348-y)
Supplement: Supplementary file 1 — Supplementary Information. [file 41598_2021_3348_MOESM1_ESM.docx]

Supplementary Information

Predicting the Side Effects of Drugs using Matrix Factorization on Spontaneous Reporting Database

Kohei Fukuto, MSc^1^; Tatsuya Takagi, PhD^1^; Yu-Shi Tian, PhD^1,^*

^1^Graduate School of Pharmaceutical Sciences, Osaka University, 1-6 Yamadaoka, Suita, Osaka 565-0871, Japan

* Corresponding author

Yu-Shi Tian

Graduate School of Pharmaceutical Sciences, Osaka University

1-6 Yamadaoka, Suita City, Osaka 565-0871, Japan

Phone No: +81-6-6879-8242

Fax No: +81-6-6879-8242

Email Address: yushi-tian@phs.osaka-u.ac.jp

Appendix: the curation of FAERS database

The FAERS database was pre-processed in the following manner. First, a dictionary was created to map the drug names to appropriate Kyoto Encyclopedia of Genes and Genomes (*KEGG)* IDs and combine multiple drug names (e.g., trade and generic names). Next, the *KEGG* IDs constituting structurally similar drugs in terms of occurrences of side effects were grouped. In the *DRUG* table, only the primary suspect drug was extracted; all duplicate drugs within the same case were removed using the *primaryid* (a unique identifier for each case in FAERS) and drug name as keys. Duplicates in the corresponding *REAC* table were also removed similarly. The pre-processed tables were then combined into a dataset that mapped drug names to their associated adverse event names. For data up to 2015 Q3, the drug names were converted into *KEGG* IDs using the *KEGG* ID dictionary and then grouped by chemical composition. Information on the adverse events of structurally similar drugs was thus summarized.

The data from 2015 Q4 onwards was reserved for independent external validation. Initially, rows containing the names of drugs that cannot be converted into *KEGG* IDs were deleted. Next, a pivot table was created that combined the Medical Dictionary for Regulatory Activities (MedDRA) preferred terms from a list of severe side effects defined by the Ministry of Health, Labour and Welfare (MHLW) into a designated side effect term^1^. The threshold was set at three or more reports to be “relevant,” and the number of reports was binarized. Then, we filtered out the drugs with less than ten side effects and vice versa. Finally, a dataset was created representing the associations between 1,127 drugs and 5,237 side effects, including 68 severe side effects.

Table S1 PR-AUC in test sets for all severe side effects with Logistic MF and other methods

|  | Logistic MF | MF | FGRMF | SVM |
| --- | --- | --- | --- | --- |
| Stevens-Johnson syndrome | 0.865 ± 0.017 | 0.877 ± 0.016 | 0.8 ± 0.023 | 0.794 ± 0.106 |
| Toxic epidermal necrolysis | 0.788 ± 0.038 | 0.808 ± 0.043 | 0.784 ± 0.027 | 0.725 ± 0.087 |
| Drug-induced hypersensitivity syndrome | 0.703 ± 0.062 | 0.708 ± 0.052 | 0.696 ± 0.078 | 0.667 ± 0.066 |
| Acute generalized exanthematous pustulosis | 0.652 ± 0.056 | 0.64 ± 0.056 | 0.609 ± 0.016 | 0.537 ± 0.066 |
| Medicament contact dermatitis | 0.732 ± 0.07 | 0.749 ± 0.031 | 0.733 ± 0.024 | 0.73 ± 0.056 |
| Erythema multiforme | 0.81 ± 0.035 | 0.805 ± 0.036 | 0.752 ± 0.042 | 0.757 ± 0.109 |
| Drug-induced liver injury | 0.801 ± 0.029 | 0.809 ± 0.011 | 0.759 ± 0.022 | 0.756 ± 0.018 |
| Acute kidney injury | 0.816 ± 0.025 | 0.805 ± 0.033 | 0.751 ± 0.048 | 0.741 ± 0.115 |
| Interstitial nephritis | 0.772 ± 0.066 | 0.761 ± 0.068 | 0.744 ± 0.093 | 0.73 ± 0.105 |
| Nephrotic syndrome | 0.794 ± 0.025 | 0.795 ± 0.026 | 0.765 ± 0.055 | 0.774 ± 0.029 |
| Vasculitis | 0.873 ± 0.028 | 0.867 ± 0.045 | 0.819 ± 0.044 | 0.736 ± 0.062 |
| Tumor lysis syndrome | 0.74 ± 0.051 | 0.694 ± 0.071 | 0.732 ± 0.028 | 0.746 ± 0.045 |
| Nephrogenic diabetes insipidus | 0.251 ± 0.138 | 0.202 ± 0.084 | 0.197 ± 0.124 | 0.116 ± 0.062 |
| Hypokalaemia | 0.913 ± 0.014 | 0.92 ± 0.013 | 0.831 ± 0.029 | 0.841 ± 0.08 |
| Aplastic anemia | 0.922 ± 0.018 | 0.93 ± 0.021 | 0.829 ± 0.054 | 0.874 ± 0.05 |
| Anemia | 0.964 ± 0.005 | 0.952 ± 0.009 | 0.84 ± 0.029 | 0.949 ± 0.051 |
| Bleeding tendency | 0.98 ± 0.006 | 0.912 ± 0.025 | 0.896 ± 0.02 | 0.983 ± 0.004 |
| Agranulocytosis | 0.936 ± 0.013 | 0.941 ± 0.008 | 0.823 ± 0.037 | 0.915 ± 0.046 |
| Thrombocytopenia | 0.948 ± 0.012 | 0.941 ± 0.021 | 0.821 ± 0.041 | 0.938 ± 0.038 |
| Thrombosis | 0.945 ± 0.012 | 0.943 ± 0.015 | 0.847 ± 0.036 | 0.929 ± 0.03 |
| Disseminated Intravascular Coagulation | 0.883 ± 0.025 | 0.891 ± 0.017 | 0.849 ± 0.037 | 0.797 ± 0.098 |
| Thrombotic Thrombocytopenic purpura | 0.736 ± 0.084 | 0.732 ± 0.068 | 0.665 ± 0.069 | 0.626 ± 0.079 |
| Heparin induced Thrombocytopenia | 0.528 ± 0.149 | 0.433 ± 0.161 | 0.372 ± 0.126 | 0.433 ± 0.153 |
| Interstitial pneumonia | 0.871 ± 0.027 | 0.878 ± 0.015 | 0.803 ± 0.033 | 0.761 ± 0.101 |
| Acute lung injury / acute respiratory distress syndrome | 0.896 ± 0.016 | 0.903 ± 0.005 | 0.811 ± 0.014 | 0.888 ± 0.011 |
| Pulmonary edema | 0.915 ± 0.025 | 0.925 ± 0.011 | 0.852 ± 0.037 | 0.906 ± 0.032 |
| Acute eosinophilic pneumonia | 0.147 ± 0.056 | 0.14 ± 0.059 | 0.137 ± 0.06 | 0.148 ± 0.079 |
| Pleuritis / pleural effusion | 0.878 ± 0.019 | 0.858 ± 0.014 | 0.822 ± 0.031 | 0.807 ± 0.066 |
| Paralytic ileus | 0.752 ± 0.059 | 0.745 ± 0.052 | 0.672 ± 0.036 | 0.689 ± 0.103 |
| Peptic ulcer | 0.87 ± 0.047 | 0.849 ± 0.057 | 0.802 ± 0.081 | 0.762 ± 0.125 |
| Pseudomembranous colitis | 0.694 ± 0.087 | 0.635 ± 0.105 | 0.619 ± 0.044 | 0.638 ± 0.057 |
| Acute pancreatitis | 0.899 ± 0.02 | 0.903 ± 0.015 | 0.844 ± 0.031 | 0.744 ± 0.112 |
| Severe diarrhea | 0.959 ± 0.011 | 0.935 ± 0.021 | 0.883 ± 0.027 | 0.921 ± 0.059 |
| Ventricular tachycardia | 0.879 ± 0.019 | 0.879 ± 0.012 | 0.82 ± 0.026 | 0.811 ± 0.119 |
| Congestive Heart Failure | 0.911 ± 0.013 | 0.906 ± 0.013 | 0.858 ± 0.028 | 0.751 ± 0.122 |
| Drug-induced Parkinsonism | 0.743 ± 0.082 | 0.713 ± 0.077 | 0.712 ± 0.083 | 0.754 ± 0.072 |
| Leukoencephalopathy | 0.685 ± 0.062 | 0.678 ± 0.079 | 0.623 ± 0.082 | 0.655 ± 0.093 |
| Rhabdomyolysis | 0.88 ± 0.023 | 0.882 ± 0.019 | 0.798 ± 0.058 | 0.81 ± 0.099 |
| Peripheral neuropathy | 0.943 ± 0.008 | 0.944 ± 0.009 | 0.886 ± 0.018 | 0.921 ± 0.04 |
| Guillain-Barré syndrome | 0.667 ± 0.071 | 0.672 ± 0.069 | 0.626 ± 0.067 | 0.651 ± 0.07 |
| Dyskinesia | 0.902 ± 0.027 | 0.899 ± 0.035 | 0.815 ± 0.078 | 0.738 ± 0.111 |
| Convulsions / Epilepsy | 0.952 ± 0.011 | 0.923 ± 0.015 | 0.857 ± 0.011 | 0.881 ± 0.061 |
| Ataxia | 0.85 ± 0.022 | 0.831 ± 0.029 | 0.781 ± 0.056 | 0.762 ± 0.074 |
| Headache | 0.976 ± 0.007 | 0.934 ± 0.011 | 0.911 ± 0.019 | 0.916 ± 0.031 |
| Aseptic meningitis | 0.543 ± 0.065 | 0.476 ± 0.062 | 0.403 ± 0.113 | 0.463 ± 0.082 |
| Acute encephalopathy in children | 0.954 ± 0.008 | 0.953 ± 0.015 | 0.845 ± 0.03 | 0.844 ± 0.105 |
| Ovarian hyperstimulation syndrome | 0.527 ± 0.473 | 0.133 ± 0.131 | 0.542 ± 0.459 | 0.506 ± 0.006 |
| Neuroleptic Malignant Syndrome | 0.771 ± 0.089 | 0.685 ± 0.078 | 0.699 ± 0.099 | 0.755 ± 0.118 |
| Drug-induced Depression | 0.948 ± 0.008 | 0.937 ± 0.013 | 0.818 ± 0.031 | 0.884 ± 0.07 |
| Akathisia | 0.792 ± 0.089 | 0.775 ± 0.079 | 0.746 ± 0.089 | 0.737 ± 0.084 |
| Serotonin Syndrome | 0.729 ± 0.064 | 0.745 ± 0.066 | 0.707 ± 0.041 | 0.683 ± 0.09 |
| Neonatal withdrawal syndrome | 0.721 ± 0.169 | 0.623 ± 0.206 | 0.717 ± 0.165 | 0.707 ± 0.171 |
| Thyrotoxicosis | 0.827 ± 0.019 | 0.835 ± 0.028 | 0.794 ± 0.06 | 0.763 ± 0.083 |
| Hypothyroidism | 0.902 ± 0.025 | 0.905 ± 0.024 | 0.862 ± 0.014 | 0.896 ± 0.032 |
| Hyperglycemia | 0.882 ± 0.02 | 0.894 ± 0.019 | 0.845 ± 0.021 | 0.895 ± 0.019 |
| Hypoglycemia | 0.891 ± 0.01 | 0.906 ± 0.016 | 0.818 ± 0.029 | 0.789 ± 0.099 |
| Anaphylaxis | 0.897 ± 0.012 | 0.904 ± 0.006 | 0.802 ± 0.031 | 0.861 ± 0.062 |
| Angioedema | 0.856 ± 0.018 | 0.868 ± 0.023 | 0.806 ± 0.039 | 0.734 ± 0.158 |
| Stomatitis medicamentosa | 0.901 ± 0.025 | 0.905 ± 0.026 | 0.875 ± 0.041 | 0.772 ± 0.119 |
| Osteoporosis | 0.886 ± 0.012 | 0.874 ± 0.012 | 0.863 ± 0.039 | 0.84 ± 0.037 |
| Idiopathic osteonecrosis of the femoral head | 0.859 ± 0.03 | 0.815 ± 0.023 | 0.798 ± 0.062 | 0.765 ± 0.072 |
| Urinary retention / Dysuria | 0.934 ± 0.017 | 0.942 ± 0.02 | 0.844 ± 0.033 | 0.872 ± 0.088 |
| Hemorrhagic cystitis | 0.525 ± 0.199 | 0.214 ± 0.037 | 0.823 ± 0.148 | 0.823 ± 0.209 |
| Glaucoma | 0.873 ± 0.016 | 0.854 ± 0.018 | 0.81 ± 0.014 | 0.857 ± 0.028 |
| Corneal opacity | 0.602 ± 0.051 | 0.367 ± 0.082 | 0.354 ± 0.046 | 0.387 ± 0.11 |
| Drug-induced hearing loss | 0.904 ± 0.024 | 0.91 ± 0.023 | 0.83 ± 0.07 | 0.921 ± 0.023 |
| Drug-induced taste dysfunction | 0.914 ± 0.022 | 0.934 ± 0.016 | 0.809 ± 0.034 | 0.892 ± 0.068 |
| Hand-Foot Syndrome | 0.727 ± 0.043 | 0.659 ± 0.036 | 0.697 ± 0.059 | 0.694 ± 0.072 |
| mean | 0.812 ± 0.021 | 0.787 ± 0.018 | 0.752 ± 0.014 | 0.763 ± 0.018 |

Table S2 PR-AUC in the external validation for all severe side effects with Logistic MF and other methods

|  | Logistic MF | MF | FGRMF | SVM |
| --- | --- | --- | --- | --- |
| Stevens-Johnson syndrome | 0.293 ± 0.013 | 0.275 ± 0.031 | 0.277 ± 0.01 | 0.133 ± 0.071 |
| Toxic epidermal necrolysis | 0.208 ± 0.018 | 0.209 ± 0.026 | 0.218 ± 0.016 | 0.151 ± 0.05 |
| Acute generalized exanthematous pustulosis | 0.297 ± 0.028 | 0.287 ± 0.045 | 0.304 ± 0.02 | 0.101 ± 0.015 |
| Medicament contact dermatitis | 0.307 ± 0.018 | 0.298 ± 0.03 | 0.313 ± 0.01 | 0.197 ± 0.058 |
| Erythema multiforme | 0.209 ± 0.014 | 0.242 ± 0.025 | 0.234 ± 0.018 | 0.143 ± 0.04 |
| Drug-induced liver injury | 0.557 ± 0.015 | 0.501 ± 0.028 | 0.527 ± 0.027 | 0.277 ± 0.049 |
| Acute kidney injury | 0.671 ± 0.007 | 0.693 ± 0.02 | 0.683 ± 0.005 | 0.489 ± 0.15 |
| Interstitial nephritis | 0.297 ± 0.013 | 0.3 ± 0.029 | 0.293 ± 0.021 | 0.179 ± 0.032 |
| Nephrotic syndrome | 0.221 ± 0.026 | 0.231 ± 0.026 | 0.241 ± 0.02 | 0.097 ± 0.049 |
| Vasculitis | 0.242 ± 0.021 | 0.214 ± 0.025 | 0.224 ± 0.017 | 0.178 ± 0.059 |
| Tumor lysis syndrome | 0.235 ± 0.019 | 0.286 ± 0.031 | 0.274 ± 0.025 | 0.11 ± 0.052 |
| Nephrogenic diabetes insipidus | 0.517 ± 0.039 | 0.198 ± 0.078 | 0.216 ± 0.074 | 0.067 ± 0.038 |
| Hypokalaemia | 0.447 ± 0.011 | 0.424 ± 0.018 | 0.459 ± 0.017 | 0.292 ± 0.08 |
| Aplastic anemia | 0.269 ± 0.031 | 0.296 ± 0.012 | 0.259 ± 0.008 | 0.276 ± 0.033 |
| Anemia | 0.28 ± 0.01 | 0.298 ± 0.018 | 0.29 ± 0.011 | 0.239 ± 0.049 |
| Bleeding tendency | 0.327 ± 0.024 | 0.335 ± 0.016 | 0.328 ± 0.025 | 0.284 ± 0.052 |
| Agranulocytosis | 0.283 ± 0.029 | 0.278 ± 0.027 | 0.253 ± 0.01 | 0.239 ± 0.046 |
| Thrombocytopenia | 0.243 ± 0.013 | 0.246 ± 0.025 | 0.264 ± 0.018 | 0.149 ± 0.017 |
| Thrombosis | 0.257 ± 0.016 | 0.261 ± 0.019 | 0.252 ± 0.02 | 0.242 ± 0.041 |
| Disseminated Intravascular Coagulation | 0.203 ± 0.032 | 0.191 ± 0.036 | 0.228 ± 0.019 | 0.141 ± 0.057 |
| Thrombotic Thrombocytopenic purpura | 0.11 ± 0.012 | 0.102 ± 0.018 | 0.092 ± 0.016 | 0.076 ± 0.054 |
| Heparin induced Thrombocytopenia | 0.132 ± 0.035 | 0.14 ± 0.098 | 0.085 ± 0.01 | 0.039 ± 0.013 |
| Interstitial pneumonia | 0.244 ± 0.016 | 0.234 ± 0.023 | 0.248 ± 0.013 | 0.2 ± 0.046 |
| Acute lung injury / acute respiratory distress syndrome | 0.318 ± 0.023 | 0.314 ± 0.025 | 0.333 ± 0.012 | 0.151 ± 0.022 |
| Pulmonary edema | 0.287 ± 0.028 | 0.283 ± 0.039 | 0.283 ± 0.024 | 0.206 ± 0.041 |
| Acute eosinophilic pneumonia | 0.097 ± 0.038 | 0.053 ± 0.035 | 0.071 ± 0.034 | 0.026 ± 0.01 |
| Pleuritis / pleural effusion | 0.331 ± 0.008 | 0.315 ± 0.011 | 0.344 ± 0.013 | 0.179 ± 0.081 |
| Paralytic ileus | 0.254 ± 0.018 | 0.214 ± 0.028 | 0.238 ± 0.016 | 0.083 ± 0.021 |
| Peptic ulcer | 0.42 ± 0.019 | 0.387 ± 0.025 | 0.402 ± 0.027 | 0.228 ± 0.106 |
| Pseudomembranous colitis | 0.184 ± 0.015 | 0.17 ± 0.017 | 0.146 ± 0.004 | 0.076 ± 0.028 |
| Acute pancreatitis | 0.43 ± 0.018 | 0.394 ± 0.033 | 0.424 ± 0.022 | 0.36 ± 0.063 |
| Severe diarrhea | 0.396 ± 0.035 | 0.421 ± 0.029 | 0.381 ± 0.032 | 0.462 ± 0.068 |
| Ventricular tachycardia | 0.287 ± 0.019 | 0.29 ± 0.022 | 0.295 ± 0.025 | 0.23 ± 0.093 |
| Congestive Heart Failure | 0.275 ± 0.02 | 0.294 ± 0.028 | 0.26 ± 0.022 | 0.159 ± 0.054 |
| Drug-induced Parkinsonism | 0.148 ± 0.006 | 0.137 ± 0.014 | 0.142 ± 0.015 | 0.091 ± 0.01 |
| Leukoencephalopathy | 0.227 ± 0.012 | 0.247 ± 0.026 | 0.259 ± 0.016 | 0.11 ± 0.066 |
| Rhabdomyolysis | 0.247 ± 0.006 | 0.291 ± 0.021 | 0.281 ± 0.009 | 0.212 ± 0.059 |
| Peripheral neuropathy | 0.392 ± 0.009 | 0.428 ± 0.012 | 0.388 ± 0.006 | 0.341 ± 0.057 |
| Guillain-Barré syndrome | 0.298 ± 0.011 | 0.247 ± 0.038 | 0.265 ± 0.044 | 0.212 ± 0.077 |
| Dyskinesia | 0.339 ± 0.013 | 0.352 ± 0.044 | 0.362 ± 0.024 | 0.307 ± 0.052 |
| Convulsions / Epilepsy | 0.21 ± 0.028 | 0.213 ± 0.03 | 0.229 ± 0.022 | 0.167 ± 0.073 |
| Ataxia | 0.317 ± 0.029 | 0.312 ± 0.043 | 0.342 ± 0.023 | 0.115 ± 0.035 |
| Headache | 0.474 ± 0.027 | 0.489 ± 0.044 | 0.43 ± 0.031 | 0.476 ± 0.087 |
| Aseptic meningitis | 0.205 ± 0.013 | 0.183 ± 0.022 | 0.184 ± 0.026 | 0.092 ± 0.034 |
| Acute encephalopathy in children | 0.35 ± 0.014 | 0.343 ± 0.021 | 0.348 ± 0.012 | 0.281 ± 0.086 |
| Ovarian hyperstimulation syndrome | 0.071 ± 0.01 | 0.04 ± 0.019 | 0.069 ± 0.033 | 0.014 ± 0.01 |
| Neuroleptic Malignant Syndrome | 0.234 ± 0.014 | 0.226 ± 0.018 | 0.251 ± 0.029 | 0.078 ± 0.067 |
| Drug-induced Depression | 0.34 ± 0.016 | 0.306 ± 0.012 | 0.285 ± 0.008 | 0.298 ± 0.045 |
| Akathisia | 0.293 ± 0.022 | 0.259 ± 0.013 | 0.281 ± 0.022 | 0.11 ± 0.103 |
| Serotonin Syndrome | 0.248 ± 0.007 | 0.239 ± 0.039 | 0.242 ± 0.018 | 0.157 ± 0.047 |
| Neonatal withdrawal syndrome | 0.211 ± 0.019 | 0.22 ± 0.05 | 0.205 ± 0.024 | 0.06 ± 0.033 |
| Thyrotoxicosis | 0.384 ± 0.016 | 0.417 ± 0.048 | 0.428 ± 0.019 | 0.185 ± 0.076 |
| Hypothyroidism | 0.342 ± 0.016 | 0.332 ± 0.016 | 0.359 ± 0.013 | 0.209 ± 0.059 |
| Hyperglycemia | 0.447 ± 0.026 | 0.437 ± 0.029 | 0.458 ± 0.017 | 0.399 ± 0.115 |
| Hypoglycemia | 0.427 ± 0.027 | 0.407 ± 0.053 | 0.427 ± 0.01 | 0.285 ± 0.11 |
| Anaphylaxis | 0.351 ± 0.013 | 0.391 ± 0.022 | 0.383 ± 0.013 | 0.322 ± 0.053 |
| Angioedema | 0.349 ± 0.011 | 0.372 ± 0.022 | 0.358 ± 0.007 | 0.213 ± 0.05 |
| Stomatitis medicamentosa | 0.334 ± 0.018 | 0.325 ± 0.029 | 0.307 ± 0.022 | 0.181 ± 0.032 |
| Osteoporosis | 0.257 ± 0.005 | 0.27 ± 0.03 | 0.285 ± 0.008 | 0.149 ± 0.014 |
| Idiopathic osteonecrosis of the femoral head | 0.201 ± 0.013 | 0.219 ± 0.032 | 0.186 ± 0.007 | 0.115 ± 0.01 |
| Urinary retention / Dysuria | 0.407 ± 0.021 | 0.405 ± 0.031 | 0.405 ± 0.016 | 0.363 ± 0.064 |
| Hemorrhagic cystitis | 0.199 ± 0.057 | 0.283 ± 0.073 | 0.291 ± 0.064 | 0.042 ± 0.021 |
| Glaucoma | 0.255 ± 0.004 | 0.253 ± 0.022 | 0.234 ± 0.017 | 0.181 ± 0.049 |
| Corneal opacity | 0.241 ± 0.009 | 0.173 ± 0.023 | 0.122 ± 0.009 | 0.059 ± 0.04 |
| Drug-induced hearing loss | 0.273 ± 0.017 | 0.299 ± 0.023 | 0.318 ± 0.019 | 0.239 ± 0.094 |
| Drug-induced taste dysfunction | 0.46 ± 0.007 | 0.427 ± 0.033 | 0.472 ± 0.012 | 0.381 ± 0.1 |
| Hand-Foot Syndrome | 0.242 ± 0.027 | 0.259 ± 0.011 | 0.282 ± 0.033 | 0.102 ± 0.02 |
| mean | 0.297 ± 0.001 | 0.291 ± 0.008 | 0.293 ± 0.002 | 0.195 ± 0.005 |

Some severe side effects were excluded due to an insufficient number of positive examples in future labels.Table S3 PR-AUC in the test set of SIDER for Logistic MF and other methods

|  | Logistic MF | MF | FGRMF | SVM |
| --- | --- | --- | --- | --- |
| Stevens-Johnson syndrome | 0.54 ± 0.085 | 0.453 ± 0.07 | 0.494 ± 0.056 | 0.689 ± 0.069 |
| Toxic epidermal necrolysis | 0.528 ± 0.083 | 0.514 ± 0.025 | 0.545 ± 0.13 | 0.584 ± 0.165 |
| Acute generalized exanthematous pustulosis | 0.097 ± 0.026 | 0.086 ± 0.072 | 0.069 ± 0.031 | 0.161 ± 0.109 |
| Medicament contact dermatitis | 0.495 ± 0.039 | 0.457 ± 0.123 | 0.467 ± 0.064 | 0.58 ± 0.114 |
| Erythema multiforme | 0.619 ± 0.064 | 0.575 ± 0.089 | 0.594 ± 0.091 | 0.744 ± 0.057 |
| Drug-induced liver injury | 0.186 ± 0.193 | 0.178 ± 0.207 | 0.064 ± 0.066 | 0.161 ± 0.203 |
| Interstitial nephritis | 0.453 ± 0.162 | 0.402 ± 0.146 | 0.448 ± 0.065 | 0.507 ± 0.053 |
| Nephrotic syndrome | 0.35 ± 0.102 | 0.303 ± 0.096 | 0.313 ± 0.06 | 0.312 ± 0.149 |
| Vasculitis | 0.494 ± 0.096 | 0.54 ± 0.069 | 0.513 ± 0.134 | 0.625 ± 0.088 |
| Tumor lysis syndrome | 0.437 ± 0.028 | 0.254 ± 0.105 | 0.404 ± 0.084 | 0.47 ± 0.155 |
| Nephrogenic diabetes insipidus | 0.05 ± 0.025 | 0.363 ± 0.451 | 0.376 ± 0.443 | 0.57 ± 0.402 |
| Hypokalaemia | 0.487 ± 0.075 | 0.517 ± 0.07 | 0.512 ± 0.022 | 0.563 ± 0.048 |
| Aplastic anemia | 0.591 ± 0.028 | 0.595 ± 0.075 | 0.638 ± 0.053 | 0.732 ± 0.047 |
| Anemia | 0.765 ± 0.031 | 0.733 ± 0.038 | 0.759 ± 0.026 | 0.87 ± 0.025 |
| Bleeding tendency | 0.757 ± 0.051 | 0.685 ± 0.049 | 0.759 ± 0.04 | 0.842 ± 0.075 |
| Agranulocytosis | 0.703 ± 0.03 | 0.674 ± 0.024 | 0.726 ± 0.042 | 0.817 ± 0.032 |
| Thrombocytopenia | 0.777 ± 0.013 | 0.742 ± 0.056 | 0.769 ± 0.03 | 0.869 ± 0.032 |
| Thrombosis | 0.556 ± 0.067 | 0.576 ± 0.043 | 0.608 ± 0.054 | 0.698 ± 0.035 |
| Disseminated Intravascular Coagulation | 0.234 ± 0.108 | 0.157 ± 0.138 | 0.136 ± 0.052 | 0.262 ± 0.256 |
| Thrombotic Thrombocytopenic purpura | 0.181 ± 0.084 | 0.103 ± 0.098 | 0.164 ± 0.14 | 0.16 ± 0.069 |
| Interstitial pneumonia | 0.344 ± 0.067 | 0.293 ± 0.036 | 0.299 ± 0.097 | 0.43 ± 0.067 |
| Acute lung injury / acute respiratory distress syndrome | 0.094 ± 0.034 | 0.124 ± 0.105 | 0.101 ± 0.086 | 0.076 ± 0.052 |
| Pulmonary edema | 0.349 ± 0.053 | 0.361 ± 0.076 | 0.361 ± 0.062 | 0.393 ± 0.086 |
| Pleuritis / pleural effusion | 0.197 ± 0.061 | 0.147 ± 0.098 | 0.178 ± 0.156 | 0.226 ± 0.077 |
| Paralytic ileus | 0.359 ± 0.123 | 0.405 ± 0.173 | 0.388 ± 0.095 | 0.424 ± 0.214 |
| Peptic ulcer | 0.453 ± 0.089 | 0.502 ± 0.101 | 0.523 ± 0.093 | 0.581 ± 0.12 |
| Pseudomembranous colitis | 0.618 ± 0.079 | 0.579 ± 0.103 | 0.612 ± 0.067 | 0.692 ± 0.074 |
| Acute pancreatitis | 0.258 ± 0.122 | 0.148 ± 0.066 | 0.158 ± 0.058 | 0.182 ± 0.097 |
| Severe diarrhea | 0.875 ± 0.024 | 0.846 ± 0.029 | 0.878 ± 0.032 | 0.903 ± 0.043 |
| Ventricular tachycardia | 0.356 ± 0.084 | 0.454 ± 0.064 | 0.492 ± 0.084 | 0.463 ± 0.049 |
| Congestive Heart Failure | 0.463 ± 0.082 | 0.423 ± 0.091 | 0.432 ± 0.073 | 0.517 ± 0.061 |
| Drug-induced Parkinsonism | 0.526 ± 0.186 | 0.547 ± 0.179 | 0.576 ± 0.1 | 0.657 ± 0.125 |
| Leukoencephalopathy | 0.421 ± 0.231 | 0.21 ± 0.162 | 0.383 ± 0.261 | 0.038 ± 0 |
| Rhabdomyolysis | 0.469 ± 0.091 | 0.454 ± 0.095 | 0.458 ± 0.131 | 0.556 ± 0.084 |
| Peripheral neuropathy | 0.504 ± 0.042 | 0.551 ± 0.067 | 0.57 ± 0.111 | 0.652 ± 0.067 |
| Guillain-Barré syndrome | 0.146 ± 0.123 | 0.061 ± 0.064 | 0.098 ± 0.041 | 0.11 ± 0.06 |
| Dyskinesia | 0.54 ± 0.098 | 0.555 ± 0.107 | 0.613 ± 0.14 | 0.619 ± 0.174 |
| Convulsions / Epilepsy | 0.677 ± 0.041 | 0.682 ± 0.04 | 0.716 ± 0.021 | 0.792 ± 0.017 |
| Ataxia | 0.64 ± 0.11 | 0.617 ± 0.053 | 0.697 ± 0.079 | 0.738 ± 0.068 |
| Headache | 0.892 ± 0.02 | 0.856 ± 0.036 | 0.925 ± 0.022 | 0.955 ± 0.017 |
| Aseptic meningitis | 0.236 ± 0.066 | 0.339 ± 0.196 | 0.426 ± 0.189 | 0.528 ± 0.18 |
| Acute encephalopathy in children | 0.443 ± 0.061 | 0.431 ± 0.069 | 0.487 ± 0.073 | 0.536 ± 0.049 |
| Ovarian hyperstimulation syndrome | 0.046 ± 0.022 | 0.108 ± 0.123 | 0.092 ± 0.081 | 0.069 ± 0.063 |
| Neuroleptic Malignant Syndrome | 0.658 ± 0.16 | 0.701 ± 0.147 | 0.722 ± 0.125 | 0.681 ± 0.113 |
| Drug-induced Depression | 0.569 ± 0.044 | 0.566 ± 0.031 | 0.623 ± 0.046 | 0.669 ± 0.066 |
| Akathisia | 0.594 ± 0.084 | 0.583 ± 0.114 | 0.605 ± 0.136 | 0.596 ± 0.112 |
| Serotonin Syndrome | 0.261 ± 0.162 | 0.28 ± 0.15 | 0.282 ± 0.131 | 0.264 ± 0.167 |
| Neonatal withdrawal syndrome | 0.469 ± 0.27 | 0.229 ± 0.147 | 0.573 ± 0.362 | 0.453 ± 0.32 |
| Thyrotoxicosis | 0.286 ± 0.095 | 0.327 ± 0.087 | 0.327 ± 0.116 | 0.388 ± 0.094 |
| Hypothyroidism | 0.501 ± 0.108 | 0.524 ± 0.17 | 0.591 ± 0.144 | 0.679 ± 0.112 |
| Hyperglycemia | 0.593 ± 0.068 | 0.624 ± 0.037 | 0.698 ± 0.051 | 0.742 ± 0.055 |
| Hypoglycemia | 0.383 ± 0.05 | 0.4 ± 0.026 | 0.431 ± 0.032 | 0.433 ± 0.12 |
| Anaphylaxis | 0.654 ± 0.037 | 0.609 ± 0.039 | 0.667 ± 0.053 | 0.724 ± 0.049 |
| Angioedema | 0.619 ± 0.033 | 0.585 ± 0.066 | 0.622 ± 0.053 | 0.733 ± 0.027 |
| Stomatitis medicamentosa | 0.598 ± 0.05 | 0.598 ± 0.057 | 0.645 ± 0.022 | 0.692 ± 0.059 |
| Osteoporosis | 0.484 ± 0.087 | 0.56 ± 0.078 | 0.529 ± 0.042 | 0.619 ± 0.061 |
| Idiopathic osteonecrosis of the femoral head | 0.489 ± 0.168 | 0.357 ± 0.161 | 0.388 ± 0.108 | 0.439 ± 0.159 |
| Urinary retention / Dysuria | 0.587 ± 0.049 | 0.611 ± 0.04 | 0.656 ± 0.057 | 0.705 ± 0.084 |
| Hemorrhagic cystitis | 0.269 ± 0.197 | 0.217 ± 0.181 | 0.104 ± 0.103 | 0.526 ± 0.014 |
| Glaucoma | 0.528 ± 0.158 | 0.485 ± 0.135 | 0.587 ± 0.117 | 0.588 ± 0.121 |
| Corneal opacity | 0.132 ± 0.086 | 0.139 ± 0.111 | 0.136 ± 0.099 | 0.149 ± 0.088 |
| Drug-induced hearing loss | 0.536 ± 0.062 | 0.463 ± 0.053 | 0.581 ± 0.062 | 0.682 ± 0.056 |
| Drug-induced taste dysfunction | 0.612 ± 0.032 | 0.617 ± 0.049 | 0.643 ± 0.042 | 0.685 ± 0.052 |
| Hand-Foot Syndrome | 0.447 ± 0.358 | 0.318 ± 0.175 | 0.541 ± 0.292 | 0.25 ± 0 |
| mean | 0.462 ± 0.015 | 0.445 ± 0.012 | 0.481 ± 0.012 | 0.551 ± 0.015 |

Some severe side effects were excluded due to an insufficient number of positive examples in future labels.


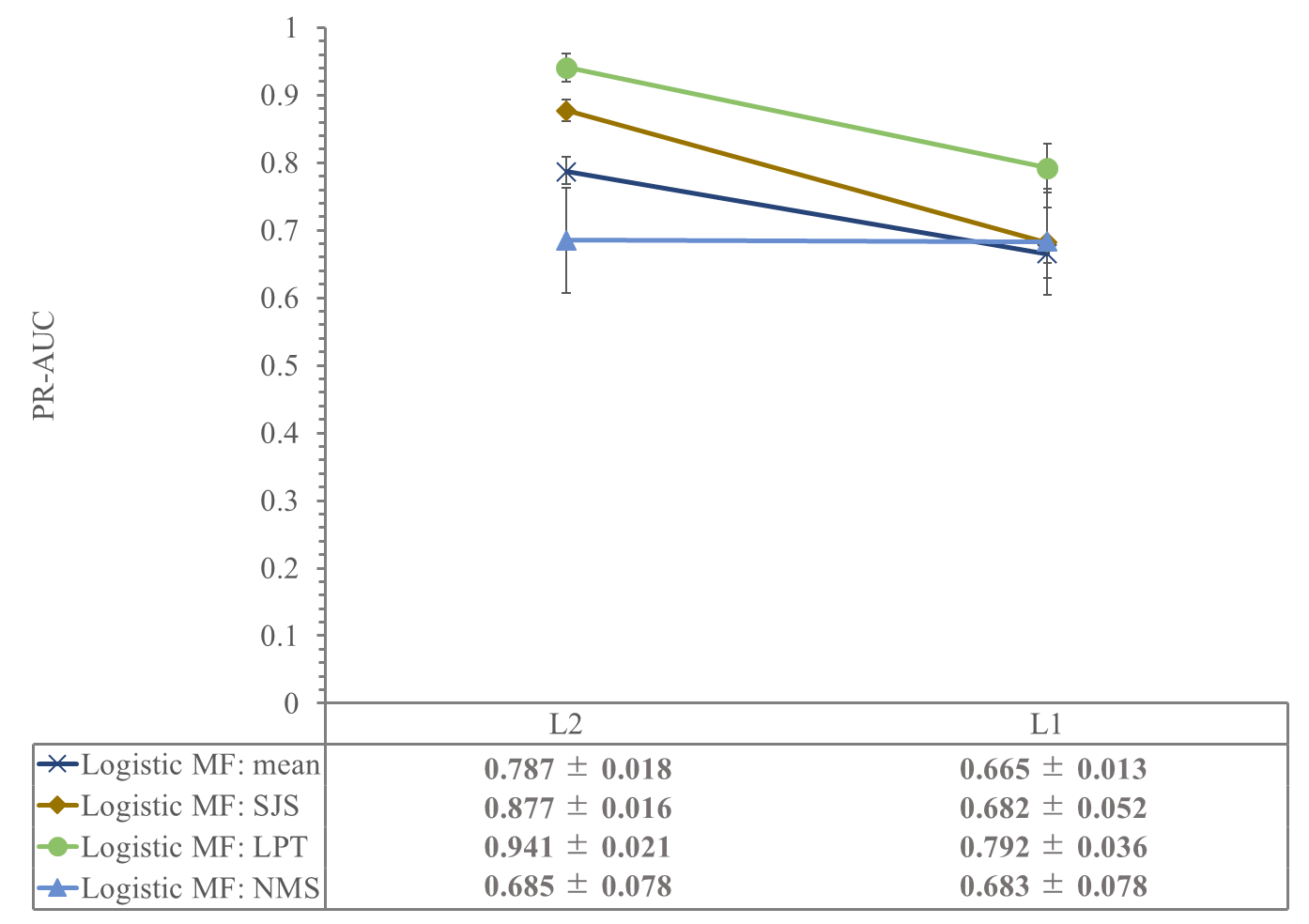


Fig. S1. Comparison of PR-AUC values using L2 and L1 norms

References

1 The list of severe side effects defined by the Ministry of Health, Labour and Welfare in Japan (source: Japanese). https://www.mhlw.go.jp/stf/seisakunitsuite/bunya/kenkou_iryou/iyakuhin/topics/tp061122-1.html. Accessed 20 Jul 2021.
